# Radiosensitisation of human colorectal cancer cells by ruthenium(II) arene anticancer complexes

**DOI:** 10.1038/srep20596

**Published:** 2016-02-12

**Authors:** R Carter, A Westhorpe, MJ Romero, A Habtemariam, CR Gallevo, Y Bark, N Menezes, PJ Sadler, RA Sharma

**Affiliations:** 1 CRUK-MRC Oxford Institute for Radiation Oncology, University of Oxford, UK; 2 Department of Chemistry, University of Warwick, UK.

## Abstract

Some of the largest improvements in clinical outcomes for patients with solid cancers observed over the past 3 decades have been from concurrent treatment with chemotherapy and radiotherapy (RT). The lethal effects of RT on cancer cells arise primarily from damage to DNA. Ruthenium (Ru) is a transition metal of the platinum group, with potentially less toxicity than platinum drugs. We postulated that ruthenium-arene complexes are radiosensitisers when used in combination with RT. We screened 14 ruthenium-arene complexes and identified AH54 and AH63 as supra-additive radiosensitisers by clonogenic survival assays and isobologram analyses. Both complexes displayed facial chirality. At clinically relevant doses of RT, radiosensitisation of cancer cells by AH54 and AH63 was p53-dependent. Radiation enhancement ratios for 5–10 micromolar drug concentrations ranged from 1.19 to 1.82. In p53-wildtype cells, both drugs induced significant G2 cell cycle arrest and apoptosis. Colorectal cancer cells deficient in DNA damage repair proteins, EME1 and MUS81, were significantly more sensitive to both agents. Both drugs were active in cancer cell lines displaying acquired resistance to oxaliplatin or cisplatin. Our findings broaden the potential scope for these drugs for use in cancer therapy, including combination with radiotherapy to treat colorectal cancer.

Some of the largest improvements in clinical outcomes for patients with solid cancers observed over the past 3 decades have been from combined treatment with chemotherapy and radiotherapy (concurrent chemo-radiation). Specifically, the addition of cisplatin and carboplatin to radiotherapy (RT) has improved prognosis for locally advanced cervical cancer, oesophageal cancer and cancer of the head and neck^1^,^2^,^3^. Chemo-radiation is therefore considered a standard treatment for these conditions. Despite the success of cisplatin for these malignancies, the addition of oxaliplatin to radiotherapy for colorectal cancer has not been shown to be of clear benefit in several large-scale studies^4^. This is particularly disappointing since chemo-radiotherapy with 5-fluorouracil is considered standard therapy for locally advanced rectal cancer prior to potentially curative surgery, and it was hoped that the synergy between oxaliplatin and 5-fluorouracil would be enhanced further by the addition of RT to chemotherapy. Unfortunately, response rates to the current international standard chemo-radiotherapy regimen (i.e. 5-fluorouracil combined with RT) for more locally advanced tumours (T3cdT4) can be as low as 38%^5^, indicating that significant numbers of patients are not successfully treated. There is, therefore, a clear need to improve radiosensitisation strategies for colorectal cancer. Moreover, cisplatin, carboplatin and oxaliplatin are associated with significant toxicities in patients with cancer, particularly when they are used in combination with RT^1^,^2^,^3^,^4^.

There is therefore considerable interest in developing other metal-based cytotoxic drugs with similar or greater anticancer activity and lower toxicity. In the last 30 years, a large number of ruthenium-containing agents have been synthesised and tested for potential anticancer activity^6^. Ruthenium (Ru) is a transition metal of the platinum group, with important differences from platinum drugs. Firstly, ruthenium(III) drugs can accumulate preferentially in cancer cells compared to normal tissues, possibly by using transferrin to enter into tumours^7^,^8^. Secondly, Ru(III) remains in its relatively inactive Ru(III) oxidation state until activation-by-reduction to Ru(II) occurs, potentially stimulated by hypoxia^9^,^10^. This may account for the apparently low toxicity of two Ru(III) agents, NAMI-A and KP1019, which have entered clinical trials in patients with cancer^11^,^12^.

The lethal effects of RT on cancer cells arise primarily from damage to DNA^4^. Cisplatin, the most commonly used agent in combination with RT in the clinic, interacts with DNA to form inter-/intra-strand cross-links, as well as DNA-protein cross-links, inhibiting DNA replication and RNA transcription and ultimately inducing mutagenesis or apoptosis^13^. In the project described here, rather than the Ru(III) coordination compounds described above, we wished to test the more reactive Ru(II) compounds in the form of organo-ruthenium, i.e. containing Ru-carbon bonds, six Ru-C bonds from an arene ring which occupies 3 of the 6 coordination positions around the pseudo octahedral Ru(II)^14^,^15^. Since organometallic Ru(II) compounds have been shown to produce complex interactions with double helical DNA^16^, we postulated that Ru(II) arene drugs may be efficacious radio-sensitisers of human cancer cells. The DNA binding activity of four of the Ru(II) arene compounds assayed in this paper: [(*η*^6^-arene)Ru^II^(en)Cl]^+^ complexes with arene = *p*-terphenyl (TB45), 1,4,9,10-tetrahydroanthracene (HC11), 9,10-dihydroanthracene (HC27), biphenyl (RM175) and indane (AH71) has been previously reported^17^,^18^,^19^,^20^,^21^. In most cases, the proposed DNA binding mechanisms involve direct coordination to a DNA base (preferentially to guanine) accompanied by intercalation of extended arenes between neighboring base pairs in the double helix. For example, such interactions appear to contribute to the high potency of TB45 towards several human cancer cell lines compared to the less active isomer AH115^17^. TB45 inhibits DNA synthesis in human ovarian cancer cells^18^. Studies of the interaction of HC11, HC27, and RM175 with plasmid DNA, ct-DNA and synthetic double-stranded polynucleotides in cell-free media^19^ have shown that DNA binding involves both coordination to guanine and hydrophobic interactions (i.e. arene intercalation and major groove binding through arene insertion). The effect of HC11 on the conformation of DNA has been studied in oligonucleotide duplexes containing a site-specific monofunctional adduct at guanine residues^20^. Even though Ru(II) arenes can bind strongly to DNA bases, they are relatively labile and can move between available binding sites, as illustrated by studies of intrastrand migration of AH71 and RM175 on a 15-mer duplex DNA^21^.

We therefore screened 14 Ru drugs to identify the 2 most promising radiosensitising agents, AH54 and AH63, which we then characterised chemically and biologically. On account of the clinical need for novel radiosensitisers for colorectal cancer described above, we focused our biological studies on human colorectal cancer.

## Results

### Identification of 2 Ru(II) complexes with significant radiosensitising activity

Organometallic ruthenium complexes of the type [(*η*^6^-arene)Ru^II^(en)Cl]^+^, offer a versatile platform for rational drug design, and have been shown to exhibit cytotoxicity that varies according to molecular structure^22^. To determine the optimal compounds to treat colorectal cancer cells in combination with radiotherapy, we measured the cytotoxicity without and with RT of 14 Ru(II) arene compounds in the colorectal cancer cell line, DLD1. This cell line was selected for the screening phase of the project since it contains a spectrum of mutations typical of colorectal cancer, including *APC*, *p53*, *KRAS* and *PIK3CA*, and because this cell line is relatively radioresistant compared to other colorectal cancer cell lines such as HCT116^23^. This rationale was consistent with a principal aim of this project, which was to identify Ru drugs which radiosensitise colorectal cancer cells currently treated inadequately by RT.

**Figure 1:**
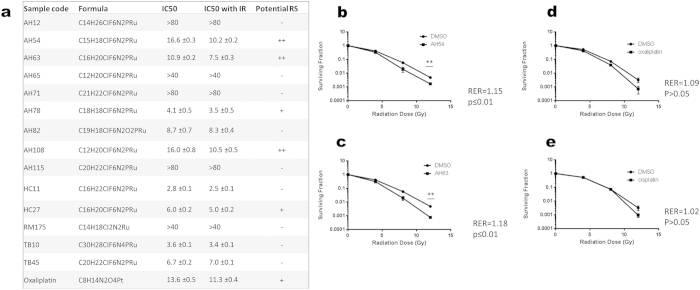
Cytotoxicity and radiosensitising activity of 14 Ru(II) compounds in DLD1 colorectal cancer cells. (**a**) IC_50_ data for Ru drugs screened without and with ionising radiation, assessed by proliferation assays. Error information indicates 95% confidence intervals. Those that displayed radiosensitising potential are marked ‘+’ for IC_50_ shift between 10 and 20% and ‘++’ for IC_50_ shift >40% (**b**–**e**) Clonogenic survival assays confirm significant radiosensitisation of DLD1 cells at 12 Gy by IC_50_ concentrations of (**b**) AH54 and (**c**) AH63, radiosensitisation by (**d**) oxaliplatin and (**e**) cisplatin does not reach significance. Statistical significances between clonogenic survival curves and radiation enhancement ratios (RER) were calculated as described in the Methods section of the main text.

Solubility of the Ru compounds was limited to ≤10 mM in DMSO, effectively limiting the maximum concentration tested to 100 μM due to DMSO toxicity at concentrations greater than 1%. Out of 14 Ru(II) compounds tested, 10 showed cytotoxicity to a degree where IC_50_ could be calculated (Fig. 1); five had cytotoxic and radiosensitisation activity at 1–50 μM, giving a degree of radiosensitisation similar to that obtained with the positive control, suberoylanilide hydroxamic acid (SAHA)^24^. Compounds AH54 and AH63 showed the best combination of low micromolar cytotoxicity and radiosensitisation effects (Fig. 1); clonogenic survival assays were used to confirm radiosensitising properties of these 2 drugs in DLD1 cells and to quantify the radiation enhancement ratio (RER) for each drug (RER ≥ 1.15).

### Chemical properties of Ru(II) complexes, AH54 and AH63

**Figure 2:**
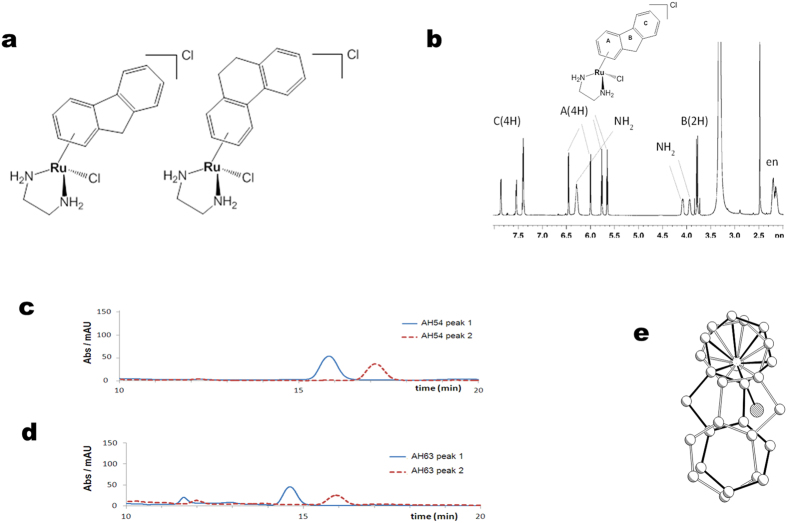
Structural properties of Ru(II) arene compounds. (**a**) Chemical structures of AH54 (left) and AH63 (right). (**b**) ^1^H NMR spectrum of AH54 in DMSO-d_6_ at 298 K. Aromatic rings within the compound are labelled A, B and C. (**c**–**d**) HPLC chromatograms showing the separated enantiomers of (**c**) AH54 and (**d**) AH63, in ethanol:heptane (30:70) with 0.5% TEA+ 0.3% TFA. (**e**) X-ray crystal structure displaying the two enantiomers of the cationic molecule of compound AH54, [(*η*^6^-flu)Ru^II^(en)Cl]^+^.

Compounds AH54 and AH63 (Fig. 2a) of general formula [(*η*^6^-flu)Ru^II^(en)Cl]^+^ (AH54; flu = fluorene) and [(*η*^6^-dihyphen)Ru^II^(en)Cl]^+^ (AH63; dihyphen = 9,10-dihydrophenanthrene), were synthesised as racemic mixtures either as Cl^−^ or PF_6_^−^ salts by the reaction of [(η^6^-arene)Ru^II^Cl_2_]_2_ dimers and the appropriate chelating ligand (i.e. en = ethylenediamine) in methanol as described previously^22^. The π-binding of the extended arene ligand to Ru(II) through one of the aromatic rings induces an asymmetry in the molecular structure of these metallodrugs. This asymmetry is reflected in their ^1^H-NMR spectra in which three sets of signals corresponding to the extended arene ligand are observed (an example is shown for AH54 in Fig. 2b).

The complexes display facial chirality, which is due to the two different orientations in which the arene ligand can bind to the ruthenium core. The arene ligand consists of biphenyl rings linked by a C-C bond and a methylene (in AH54) or an ethylene (in AH63) bridge in the ortho position, which forms a third central five-membered or six-membered ring, respectively. This central ring formation forces the whole arene ligand to become coplanar with the central ring adopting either syn- or anti- configuration, which in turn allows for the generation of two enantiomers for both compounds. Thus the formation of enantiomers in this case depends on the coordination of the arene to the ruthenium centre through either arene face.

Chiral HPLC was employed in order to separate the enantiomers. Two peaks were obtained corresponding to the two enantiomers for each compound, which were then isolated and shown to be stable with regard to racemization over a very long period of time (over a year, *vide infra*), Fig. 2c,d. The structure of facially chiral complex AH54 had been previously determined by X-ray diffraction; the crystal structures of the two enantiomers for AH54 are shown in Fig. 2e^22^.

**Figure 3:**
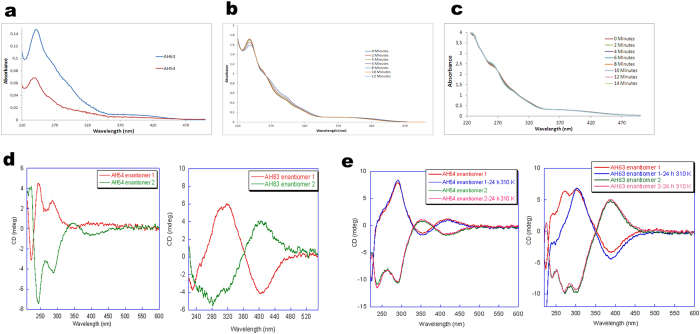
Stability of AH54 and AH63 in solution. (**a–c**) Ultraviolet-visible (UV-vis) spectroscopy of AH54 and AH63 to determine electronic transitions bands and aquation times. (**a**) UV-vis spectra of AH54 and AH63 in ethanol. Broad bands at ~240 nm can be attributed to π-π* transitions in the arene ligands. (**b**) UV-vis spectra showing aquation time of AH54 in PBS (1 mM, pH 7.2). A change on the intensity of the initial bands and an isosbestic point at ~270 nm indicate replacement of Cl^−^ in the compound by water. (**c**) UV-vis spectra showing aquation time of AH63. (**d**) Complementary circular dichroism (CD) spectra confirm that the HPLC–separated enantiomeric fractions correspond to mirror images of the chiral complexes. (**e**) Configurational stability of the enantiomers in PBS (1 mM, pH 7.4) is demonstrated by the lack of significant change in CD spectra after 24 hours of incubation at 310 K for AH54 and AH63.

UV-Vis spectra were recorded for AH54 and AH63 in ethanol (Fig. 3a) in order to identify their characteristic electronic absorption bands. Both compounds show an intense band at ca. 240 nm, which is attributed to π→π* transitions from the arene ligands. This broad band exhibits a shoulder at ca. 260 nm. Additionally a low intensity absorption band was observed for both complexes at ca. 400 nm, which can be assigned to metal-centred transitions. Aquation of these compounds, by which the active form of the complexes is generated, occurred in a short period of time, with AH54 becoming fully aquated within 12 minutes and AH63 within 14 minutes (Fig. 3b,c) at 310 K, as confirmed by Uv-vis and NMR spectroscopy. When dissolved in 150 mM NaCl solution, aquation was suppressed, due to an increased concentration of Cl^-^ anions inhibiting the hydrolysis of the Ru-chloride bond.

Circular dichroism spectra (CD) were recorded in order to confirm the enantiomeric nature of the two fractions of complexes AH54 and AH63 separated by HPLC. This technique determines the differential absorption of left- and right- circularly polarized light and is widely employed for the study of chiral molecules. However it cannot provide information on the absolute configuration at the chiral metal centre in these complexes. The ruthenium complexes AH54 and AH63 did not exhibit CD signals as expected for racemic mixtures. However the enantiomer fractions isolated for each complex gave complementary CD spectra, thus confirming the two fractions correspond to mirror images of the planar chiral complexes (Fig. 3d). In order to determine the stability of each isolated enantiomer in solution, they were re-injected into the HPLC 24 h after isolation and the chromatograms showed no significant differences to those seen initially, suggesting that the enantiomers were stable and did not undergo racemization. The configurational stability of each enantiomer after incubation for 24 h under biological relevant conditions (1 mM PBS, pH 7.4, 310 K) was also confirmed by circular dichroism (CD) spectroscopy (Fig. 3e).

### Cytotoxicity of Ru complexes in DNA damage repair-deficient cell lines

**Figure 4:**
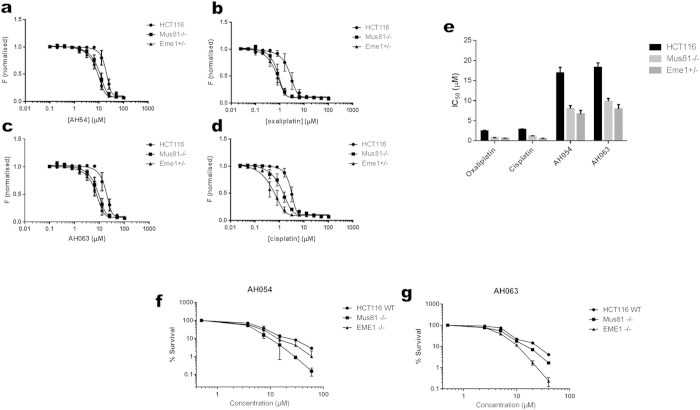
Ruthenium compounds exhibit greater cytotoxicity in cell lines deficient in DNA damage repair. Cytotoxicity was compared for HCT116 (parental) and the isogenic DNA damage repair-deficient (Mus−/− and Eme1 +/−) HCT116 variants. IC_50_ curves were produced from cell proliferation assay data and the separation between the IC_50_ curves was indicative of the effect of Mus81 or Eme1 status on drug response. (**a–d**) IC_50_ curves for wildtype, Mus81 −/− and Eme1 +/− HCT116 strains following exposure to (**a**) AH54, (**b**) oxaliplatin, (**c**) AH63 or (**d**) cisplatin; p ≤ 0.05 for each compound tested. (**e**) Summary of IC_50_ values for the compounds tested. (**f–g**) Confirmation by clonogenic survival assays for (**f**) AH54 and (**g**) AH63.

There are several independent cellular mechanisms of action by which Ru drugs can cause cytotoxicity to cancer cells^25^,^26^,^27^. In order to demonstrate that the cytotoxicity of AH54 and AH63 is related to DNA damage induced by these drugs, we studied their effect on colorectal cancer cells lacking the ability to repair DNA damage. We chose to study isogenic HCT116 colorectal cancer cells deficient in DNA damage repair proteins, Mus81 and Eme1, since these proteins have been shown to be involved in inter-strand crosslink repair^28^, repair of erroneous replication forks^29^ and Holliday junction resolution^30^. Importantly, these proteins are not known to have any cellular functions other than DNA damage repair. It has also been shown that Eme1-null cells are hypersensitive to cisplatin, suggesting the role of this repair protein in rescuing the cell from chemotherapeutically-induced DNA damage^31^. As shown in Fig. 4, we demonstrated by proliferation assays and clonogenic survival assays that both Ru drugs were significantly more cytotoxic in cells deficient in these DNA damage repair proteins than in parental HCT116 colorectal cancer cells (p ≤ 0.05 by paired Student’s T test), consistent with the hypothesis that DNA damage induced by both drugs is related to their cytotoxicity in colorectal cancer cells.

### p53 status determines cell cycle arrest and apoptosis following treatment with AH54 and AH63

p53 is an essential tumour suppressor protein and a central mediator of cellular responses to DNA damage and other stresses^32^. We chose to study the role of p53 status in determining the cytotoxicity of AH54 and AH63 in colorectal cancer cells on account of the importance of p53 to colorectal cancer^33^, the central role of p53 in dictating radiosensitivity^34^ and previous observations that the effects of certain arene-Ru(II) diamines on cancer cells can be influenced by p53 status^35^,^36^.

We tested the effect of p53 status on response to AH54 and AH63 using isogenic HCT116 cells that were either p53 wildtype (+/+), p53 null (−/−), or p53 mutated (S248W/-) carrying a common cancer mutation in the DNA binding domain of the p53 protein. Cell cycle effects were apparent between 12 h and 48 h. Only the p53 wildtype cells exhibited significant cell cycle arrest in G2/M phase (Supplementary figure S1a); p53 mutated cells showed little effect and the p53-null cells replicated their DNA as normal, proceeding to endo re-duplication after 48 hours, as their genome doubled, but the cells failed to divide (Supplementary Figure S1b).

**Figure 5:**
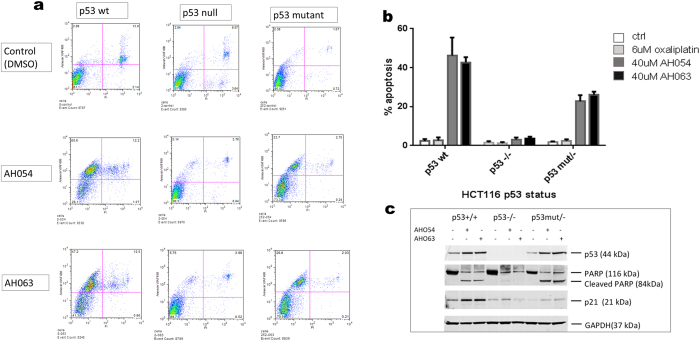
HCT116 cells treated with IC_80_ of AH54 or AH63 display apoptosis only when p53 status is wildtype or mutated. (**a**) FACS analysis showing Annexin versus propidium iodide (PI) staining. (**b**) Summary of apoptosis data obtained from 3 independent experiments. (**c**) Western blot showing PARP cleavage consistent with apoptosis (full length blots are presented in Supplementary Figure S2).

Ru(II) arene compounds have previously been reported to induce apoptosis in cancer cells^35^,^36^. We compared the apoptosis caused by AH54 and AH63 at IC_80_ with apoptosis caused after treatment with oxaliplatin at IC_80_. Treated cells were stained for Annexin V, to identify both apoptotic and necrotic cells, and propidium iodide, which stains necrotic cells due to their permeabilised membrane. The percentage of apoptotic cells was determined from a plot of Annexin V staining versus propidium iodide staining. As shown in Fig. 5, after 24 h of oxaliplatin treatment, apoptosis was similar to the negative control (DMSO) for each p53 variant of HCT116. AH54 and AH63 caused comparable levels of apoptosis; >40% in the p53 wildtype cells, >20% in the p53 mutated cells, and low levels in the p53-null cells (<4%; slightly higher than background).

These results show that these organo-ruthenium compounds induce significant apoptosis in colorectal cancer cells and that this effect depends on the p53 status of the cells. To confirm these observations, we measured protein levels in extracts of cells treated with IC_80_ concentrations of each drug. Western blots confirmed p53 stabilisation following drug treatment in both the p53 wildtype and mutated cell lines, with PARP cleavage also occurring in both (Fig. 5c and Supplementary Figure S2). However p21 induction was observed only in the p53-wildtype cells, confirming the lack of a functional p53 pathway in the p53-mutated variant.

### Radiosensitisation by AH54 and AH63 is dependent on p53 status of cancer cells

**Figure 6:**
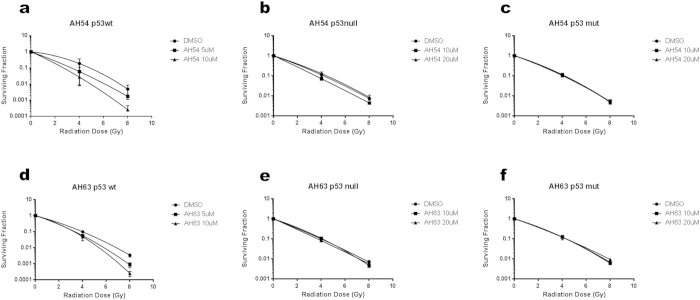
Radiosensitisation by AH54 and AH63 is dependent on the p53 status of the cell. (**a–c**) AH54 induces dose-dependent radiosensitisation exclusively in p53 wildtype HCT116. Survival curves of HCT116 cells with p53 status (**a**) wildtype, (**b**) null or (**c**) mutated (RER at 5 μM AH054 = 1.28, at 10 μM AH54 = 1.58). (**d–f**) Survival curves of HCT116 cells treated with AH63, p53 status is (**d**) wildtype, (**e**) null or (**f**) mutated (RER at 5 μM AH63 = 1.18, at 10 μM AH63 = 1.26).

It was noted that the magnitude of the radiosensitisation effect observed in DLD1 cells (Fig. 1) was not particularly marked, with statistically significant separation of the clonogenic survival curves occurring at doses ≥6 Gy. We postulated that this lack of effect at lower radiation doses per fraction was due to the mutant p53 status of the DLD1 cells, which are known to be relatively radioresistant compared to HCT116 cells^23^. As fraction sizes of 1.8 to 5 Gy are used to treat rectal cancer in the clinic, the effect of p53 status in more radiosensitive HCT116 colorectal cancer cells was also studied in the dose range 0–8 Gy. By clonogenic survival assay, a supra-additive radiation enhancement effect was demonstrated by the combination of each drug with radiation in the p53 wildtype cells (Fig. 6 and Supplementary Figure S3). The radiation enhancement ratio (RER) was measured at surviving fraction 0.1 (RER = 1.46 for 5 μM AH54, RER = 1.82 for 10 μM AH54, RER = 1.19 for 5 μM AH63, RER = 1.22 for 10 μM AH63). The same doses of drug and radiation fraction size did not result in significant radiosensitisation of p53-null and p53-mutant variants of the same cell line. These results suggest that AH54 and AH63 may be used to radiosensitise p53-wildtype colorectal cancer at RT fraction sizes used clinically.

### AH54 and AH63 are effective against oxaliplatin-resistant and cisplatin-resistant cancer cells

**Figure 7:**
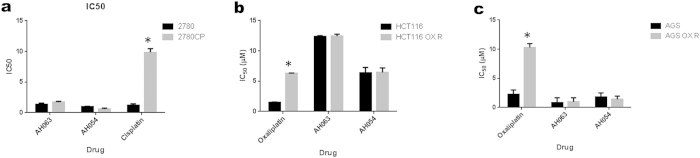
Activity of compounds AH54 and AH63 in platinum-sensitive and platinum-resistant human cancer cell lines. (**a**) IC_50_ of Ru drugs and cisplatin in cisplatin-sensitive (A2780) and cisplatin-resistant (A2780CP) ovarian cancer cells. (**b**) IC_50_ of Ru drugs and oxaliplatin in isogenic oxaliplatin-sensitive (HCT116) and oxaliplatin-resistant (HCT116 OX R) colorectal cancer cells. (**c**) IC_50_ of Ru drugs and oxaliplatin in oxaliplatin-sensitive (AGS) and oxaliplatin-resistant (AGS OX R) gastric cancer cells. For all experiments shown above, IC_50_ was measured by cell proliferation assay and treated values were normalised to those of untreated control wells. Using Student’s T test, significant differences were noted between cisplatin-resistant and cisplatin-sensitive A2780 cells treated with cisplatin (p ≤ 0.05 using paired Student’s T test), but no significance between the same cells treated with AH54 and AH63 (Panel A). The same level of significance was observed in oxaliplatin-resistant and oxaliplatin-sensitive HCT116 and AGS cells treated with oxaliplatin (Panels b and c), with no significant difference in IC_50_ between the same cells treated with AH54 and AH63.

To investigate the potential utility of AH54 and AH63 for the treatment of human cancers that have acquired oxaliplatin and cisplatin resistance, we measured the IC_50_ values of the Ru compounds in isogenic platinum-sensitive and -resistant variants. AH54 and AH63 were compared to oxaliplatin in the oxaliplatin-sensitive and resistant AGS (gastric cancer) and HCT116 (colorectal cancer) cell lines and to cisplatin in cisplatin-sensitive and cisplatin-resistant A2780 (ovarian cancer) cells. As expected, the IC_50_ values for cisplatin and oxaliplatin were up to 10-fold lower in the sensitive cell lines than in the resistant (Fig. 7). The IC_50_ values of the Ru compounds were similar between both the platinum-sensitive and platinum-resistant cell lines, indicating no cross-resistance between the Ru drugs and the platinum drugs.

## Discussion

The ruthenium complexes tested in this project were designed to be cytotoxic to cancer cells, having a planar arene structure to intercalate with DNA and chlorido-ligands to optimise aquation and direct binding to DNA bases (especially guanine). This is the first study to show that Ru(II) arene drugs are radiosensitisers when used in combination with clinically–relevant doses of RT. The findings presented here broaden the potential scope for these drugs to be developed for cancer therapy, including potential advancement to clinical trials in patients who require radiotherapy as standard treatment for colorectal cancer.

Our chemical results suggest that the Ru complexes AH54 and AH63 undergo rapid aquation at physiological pH. The complexes are isolated as racemic mixtures of enantiomers which exhibit facial chirality. The HPLC chiral separation of each enantiomer of both complexes allowed us to analyse their CD spectra (in ethanol), which showed several absorption bands with maximum intensities in the range 220–350 nm (Fig. 3d). These bands may be attributed to π→π* transitions of the coordinated arene ligand. An additional band was observed between 350–500 nm due to metal-based transitions. Complementary CD spectra were obtained for the HPLC-separated enantiomers, thus confirming that the isolated pseudo-octrahedral chiral complexes are mirror images of each other. This planar (facial) chirality is induced by the syn- or anti- configuration adopted by the central arene ring due to the asymmetric nature of the capping arene when binding to ruthenium(II) during metal complex formation. The separated enantiomers were stable in solution and did not show a tendency towards racemization, as demonstrated by HPLC and CD configurational stability studies (Fig. 3e) showing the configurational status did not change after 24 h in solution. It is possible that the two enantiomers may demonstrate differential DNA binding activity worthy of further investigation, but at the present time it has not been possible to obtain sufficient purified quantities of these compounds for biological testing.

Several of the ruthenium compounds tested had cytotoxicity at low micromolar concentrations in DLD1 colorectal cancer cells. In general, the cytotoxicity against cancer cells of the ruthenium(II) metallodrugs studied increases with extension of the arene ring or with an increase in the lipophilicity of the *N, N-*chelating ligand (Fig. 1a and Supplementary Table S1). The increase of hydrophobicity is likely to favour the cellular uptake of these complexes. An enhancement of the anticancer activity of Ru(II) arene complexes is observed when the aliphatic *N, N-*chelating ligand ethylenediamine (en) is replaced by an aromatic *N, N-*chelating ligand while maintaining the same arene, as illustrated by the activity of AH12 versus TB10 (arene = hexamethylbenzene), AH71 compared to AH82 (arene = indane), and RM175 compared to AH78 (arene = biphenyl).

For the Ru(II) en complexes, the number and orientation of phenyl ring substituents on the arene ligand play an important role (Fig. 1a and Supplementary Table S1). For example, the cytotoxicity increased up to 6x when the phenyl group was incorporated at the *para* position with respect to the bound arene (e.g. RM175 versus TB45). The presence of fused arene rings in HC11 and HC27 resulted in higher cytotoxic activity compared to the more compact arene in AH63 (HC11, arene = 1,4,9,10-tetrahydroanthracene; HC27, arene = 9,10-dihydroanthracene; AH63, arene = 9,10-dihydrophenanthrene). Moreover complex AH71 which has a 5-membered cyclic ring fused onto the arene ligand (i.e. indane) is less active than complex AH108 bearing a 6-membered cyclic ring fused onto the arene, perhaps due to a change in lipophilicity.

In cell proliferation assays for 5 of the Ru drugs, AH54, AH63, AH78, AH108 and HC27, IC_50_ curves normalised for radiation effect were significantly different, indicating a degree of radiosensitisation. With large fractions of RT, radiosensitisation was confirmed in DLD1 cells for AH54 and AH63 by clonogenic survival assays.

In HCT116 colorectal cancer cells, there was no additive effect (radiosensitisation) observed in mutant or null p53 variants for either compound; both AH54 and AH63 caused dose-dependent radiosensitising activity in wildtype p53 cells. It is recognised that p53 is responsible for responding to DNA damage caused by ionising radiation and other DNA-damaging treatments^37^ and inducing cell cycle arrest and apoptosis in the affected cells^34^. In confirmation of this, we observed cell cycle arrest in G2/M phase after treatment with AH54 and AH63 in the p53-wildtype cells, but not in the p53-null or p53-mutant variants. As G2 and M phases of the cell cycle are more radiosensitive than other phases of the cell cycle^38^, this finding is a potential explanation for the significant radiosensitisation observed.

Apoptosis occurred in both the p53-wildtype and p53-mutant HCT116 cells, which is encouraging as it indicates that these drugs may be effective in tumours in which p53 is mutated^32^. This observation is also consistent with the radiosensitisation observed in p53-mutant DLD1 cells treated with large fractions of RT. These results in p53-wildtype cells are consistent with a previous study of a ruthenium complex containing a bis-benzimidazole derivative, which showed radiosensitising ability and G2/M cell cycle arrest in p53-wildtype A375 melanoma cells^39^,^40^. Our results, particularly in view of the chiral stability that we have demonstrated, identify AH54 and AH63 as promising radiosensitisers, particularly in cancer cells with wildtype p53 status.

The effects of AH54 and AH63 we have shown in cisplatin-resistant and oxaliplatin-resistant cell lines are consistent with our previous findings using other Ru(II)-arene compounds tested in cisplatin-resistant cells^22^,^41^. These data may be relevant to the use of RT in patients with colorectal cancer who have previously received oxaliplatin-based chemotherapy and whose tumours have acquired chemotherapy resistance. Our results suggest that compounds should be equally effective in this clinical scenario, with no evidence of cross-resistance to platinum agents.

The potential for Ru(II)-arene compound activity i*n vivo* has been shown previously with RM175 tested in a xenograft model with A2780 ovarian cancer cells, and cisplatin-resistant A2780 cells^41^. The complex [(η^6^-fluorene)Ru(en)Cl]PF_6_ (AH54) has been synthesized with incorporation of the β-emitting radioisotope ^106^Ru (half-life = 1.01 y). Distribution studies 0.25 h post i.v. injection of ^106^Ru-1 at a dose of 25 mg kg^−1^ showed that ^106^Ru is well distributed throughout the tissues of a rat^46^. Furthermore, the related organometallic osmium arene complex, [Os(η^6^-*p*-cym)(4-(2-pyridylazo)-N,N-dimethylaniline)I]PF_6_, has been shown to delay the growth of HCT116 human colon cancer xenografts in mice, with negligible toxicity^47^.

It has been suggested that some ruthenium agents may demonstrate greater efficacy against cancer metastases than against primary tumors^42^. This antimetastatic effect may be mediated by inhibition of tumour cell detachment, invasion/migration, and re-adhesion to a new growth substrate^43^. Such a phenomenon has not previously been observed with drugs currently used in combination with RT (cisplatin or 5-fluorouracil), and it is relevant to new forms of large-fraction radiotherapy now available for treating metastases from colorectal cancer^44^,^45^.

In summary, we have characterised the chiral Ru(II)-arene complexes AH54 and AH63 in terms of chemical structure and biological functionality in treating human colorectal cancer cells. We have shown significant radiosensitisation with clinically–relevant doses of RT, greater radiosensitising activity in p53-wildtype cells compared with p53-null or p53-mutated, and a biological mechanism of action that appears to involve DNA damage induced by these drugs. If these results are confirmed in *in vivo* preclinical models, we propose that these agents should be developed further as anticancer agents in combination with RT.

## Materials and Methods

### Synthesis of Ru compounds

All complexes were synthesized using a similar procedure. Typically the ligand (2 mol equiv.) was added to a methanolic solution of the dimer [(*η*^6^-arene)RuCl_2_]_2_.

The following compounds were synthesized as described in the literature:

**AH12, AH54, AH63, AH65, AH71, AH78, AH82, AH108 and TB10**^22^, **HC11 and HC27**^48^**, TB45 and AH115**^17^**, RM175**^49^.

### Further characterization of compounds AH54 and AH63, studied in detail in this work, is provided below


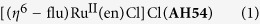


^1^H NMR (400 MHz, MeOD-d_4_, ppm): 7.87 (m, 1H), 7.59 (d, *J* = 7.1 Hz, 1H), 7.50–7.42 (m, 2H), 6.35 (d, *J* = 5.7 Hz, 1H), 6.15 (s, 2H, NH_2_), 6.04 (d, *J* = 5.7 Hz, 1H), 5.93 (s, 2H, NH_2_), 5.77 (t, *J* = 5.5 Hz, 1H), 5.71 (t, *J* = 5.4 Hz, 1H), 3.96 (d, *J* = 21.8 Hz, 1H), 3.81 (d, *J* = 21.8 Hz, 1H), 2.55–2.24 (m, 4H); Uv-vis (λ_máx_, nm, EtOH): 240, 262 (sh), 398.

**1**-enantiomer 1: HPLC (t_r_, 254 nm) = 15.7 min; CD (EtOH; λ_max_, nm (Δε, mol^−1^cm^−1^)): 222 (−3), 244 (+4), 266 (+2), 286 (+3), 349 (+0.03), 402 (+0.4).

**1**-enantiomer 2: HPLC (t_r_, 254 nm) = 17.1 min; CD (EtOH; λ_max_, nm (Δε, mol^−1^cm^−1^)): 222 (+4), 244 (−7), 266 (−3), 286 (−4), 349 (+0.5), 402 (−0.6).


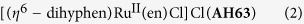


^1^H NMR (400 MHz, MeOD-d_4_, ppm): 7.81 (dd, *J*_*1*_ = 7.5 Hz, *J*_*2*_ = 0.9 Hz, 1H), 7.43 (dt, *J*_*1*_ = 7.4 Hz, *J*_*2*_ = 7.4 Hz, *J*_*3*_ = 1.1 Hz, 1H), 7.36 (t, *J* = 7.3 Hz, 1H), 7.32 (d, *J* = 7.4 Hz, 1H), 6.34 (s, 2H, NH_2_), 6.17–6.13 (m, 1H), 5.82–5.73 (m, 3H), 5.72–5.68 (m, 1H), 4.04 (s, 1H, NH_2_), 3.88 (s, 1H, NH_2_), 2.95–2.82 (m, 2H), 2.78–2.65 (m, 1H), 2.64–2.52 (m, 1H), 2.46–2.28 (m, 3H); Uv-vis (λ_máx_, nm, EtOH): 243, 262 (sh), 396.

**2**-enantiomer 1: HPLC (t_r_, 254 nm) = 14.7 min; CD (EtOH; λ_max_, nm (Δε, mol^−1^cm^−1^)): 297 (+5), 318 (+6), 403 (−4).

**2**-enantiomer 2: HPLC (t_r_, 254 nm) = 16.0 min; CD (EtOH; λ_max_, nm (Δε, mol^−1^cm^−1^)): 285 (−5), 318 (−4), 403 (+4).

### Chemical analysis

#### pH* measurements

pH* values (pH meter reading from D_2_O solution without correction for effect of deuterium on glass electrode) were measured at ambient temperature before the NMR spectra were recorded, using a Corning 240 pH meter equipped with a microcombination electrode calibrated with Aldrich buffer solutions at pH 4, 7 and 10.

#### UV-vis Spectroscopy

UV-vis spectra were recorded on a Cary 300 spectrophotometer using 1-cm path-length quartz cuvettes (0.5 mL) and a PTP1 Peltier temperature controller. Spectra of the Ru(II) arene complexes were recorded in EtOH (0.01 mg/mL) at 298 K from 800 to 200 nm. Kinetic measurements were made at a Ru(II) complex concentration of 0.1 mM in PBS (1 mM, pH 7.4) at 310 K and the spectra were recorded in intervals of 2 min for 5 h. All data processing was carried out using Excel 2007 or Kaleidagraph.

#### NMR Spectroscopy

^1^H NMR spectra were acquired in 5 mm NMR tubes at 298 K on a Bruker DPX-400 spectrometer, using DMSO-d_6_, MeOD-d_4_ or PBS in D_2_O solution (1 mM, pH* 7.4) as deuterated solvents and 1,4-dioxane as reference. All data processing was carried out using MestReC or TOPSPIN version 2.0 (Bruker U.K. Ltd.).

#### HPLC

Semi-preparative separation of the enantiomers was carried out with the following HPLC equipment: two Gilson 306 pumps, a Gilson 805 manometric module, a 811 dynamic mixer, a 234 autoinjector and Hewlett Packard 1100 Series DAD Detector. The data were analysed using UniPoint Version 5.11 Software. Each complex was dissolved at a concentration of 0.6 mg/mL (AH63) and 1 mg/mL (AH54) in ethanol (HPLC grade) prior to chromatographic separation. The enantiomer separation was carried out on a semi-preparative Chiralpack IC (25 cm length x 1 cm inner diameter, 5 μm) column (Chiral Technologies Europe) at a detection wavelength of 254 nm. Operation conditions: normal phase, isocratic elution (mobile phase heptane:ethanol (70:30) in 0.5% TEA (triethylamine) + 0.3% TFA (trifluoroacetic acid), injection volume 450 μL, flow rate 2.5 mL/min, T = 295 K). The collected fractions were concentrated under vacuum to dryness and storage at 253 K. The characterization of the enantiomers was carried out on an analytical Chiralpak IC (25 cm length x 4.6 mm inner diameter, 5 μm) column (Chiral Technologies Europe) using the same mobile phase and isocratic elution. Operation conditions: injection volume 50 μL, flow rate 0.5 mL/min, T = 295 K).

#### Circular dichroism

Circular dichroism spectra of AH54 and AH63 were recorded in the range 700–200 nm on a J-815 circular dichroism spectropolarimeter (Jasco, UK) at ambient temperature. The spectra were obtained for all the samples in ethanol (HPLC grade) or PBS (1 mM, pH 7.4) using a quartz cuvette of 0.1 cm path-length, scan speed 200 nm/min, 3 accumulation scans, 1 nm band width, 0.2 nm data pitch and 0.5 s of response time.

#### Cell culture and treatments

HCT116 cells isogenic for p53 status were a gift from Dr Bert Vogelstein (Johns Hopkins School of Medicine, Baltimore, USA). Variants used were p53 wildtype (parental strain), p53 null (both alleles inactivated by deletion of exons 2–4 through targeted homologous integration) and p53 mutated (allele 1 mutated by knock-in of R248W mutation in exon 7, allele 2 inactivated by deletion of exon 2).

Additional HCT116 isogenics (Mus81 null and Eme1 haplo-insufficient) were generated by gene targeting^50^ and were a gift from Prof. Kiyoshi Miyagawa (University of Tokyo, Japan).

Platinum–resistant and sensitive variant cell lines were developed by repeated exposure to stepwise-increasing concentrations of oxaliplatin or cisplatin over a period of up to 10 months. Ovarian cancer cell line A2780, and cisplatin-resistant variant^51^ were obtained from Dr Euan Stronach (Imperial College, London). Gastric cancer cell line AGS and oxaliplatin-resistant variant were a gift from from Dr S. Madhusudan (University of Nottingham, UK). Oxaliplatin-resistant and -sensitive HCT116 cells (Boyer, McLean, Aroori, Wilson, 2004) were a gift from Dr. P. Johnston (Queen’s University Belfast, UK). To maintain resistance, cells were treated every 3–6 passages with 1 μM cisplatin (A2780CP), 5 μM oxaliplatin (AGS OXR) or 8 μM oxaliplatin (HCT116 OXR). Cells were cultured at 37 °C in a 5% CO_2_, humidified incubator. Culture media used was RPMI for A2780 and AGS, McCoy’s modified GlutaMAX for p53 isogenic HCT116 and DMEM for HCT116 OXR, each supplemented with 10% foetal calf serum and 1x penicillin streptomycin.

To determine whether drugs acted synergistically with radiation, cells were either mock-irradiated or treated sequentially with drugs and radiation. Plates were irradiated using a Gamma-Service Medical GmbH GSR D1 irradiator containing a caesium-137 source at room temperature, at a dose rate of 1.5 Gy/min.

### Cell proliferation assays

Cells were plated on the central 60 wells of a 96-well plate with 1,000–3,000 cells/ well (depending on plating efficiency) in 200  μl media. Cells were rested overnight, and drugs added in serial dilution, in triplicate. For drug-radiation combinations, plates were either mock irradiated or irradiated 6 hours following drug treatment. After 24 hours, drugs were replaced with fresh media, and cells incubated for 5 cell division times. The effect of the drug and radiation treatments was quantified by using resazurin, a metabolically-activated dye. This experimental procedure has been demonstrated to correlate linearly with cell number, and gives results comparable to the data obtained from clonogenic assays. Medium was replaced by phenol red-free DMEM containing 10 μg/ml resazurin, and incubated at 37 °C for 2–4 hours, resazurin reduction was measured via fluorescence at 590 nm using a BMG POLARstar Omega plate reader (BMG Biotech, Aylesbury, UK).

### Clonogenic survival assays

Clonogenic survival assays were on 6-well plates, with two replicates of each variable. Cells were plated in serial dilution according to radiation dose, with one log increase in cell number/ 4 Gy radiation. Cells were allowed to attach for at least 6 hours before drug addition, and irradiated 6 hours after drug addition, as described. Medium was refreshed 24 hours after radiation, and surviving cells allowed to proliferate for 10–14 days. Cells were fixed for 30 minutes in 0.4% methylene blue dye in methanol. Colonies of more than 50 cells were counted, and the surviving fraction (SF) calculated from the ratio of colonies observed/ expected. Radiation survival curves were fitted using the linear–quadratic model; α and β values were calculated at 4 Gy and 8 Gy using the equation SF = e^(α*D + β*D^2); each fitted curve represents the mean of at least 3 independent experiments.

### FACS analysis

For cell cycle analysis, 10^6^ cells/ well from exponentially growing cultures were plated on 6–well plates, rested overnight and treated with compounds AH54 and AH63 at IC_50_ or IC_80_ concentrations. At intervals up to 48 hours after drug treatment, cells were trypsinised, PBS washed, and fixed in 70% ice cold ethanol. After PBS wash, DNA was labelled with 10 μg/ml propidium iodide, and PI staining measured using a Becton-Dickinson FACScan. For apoptosis assays harvested cells were stained with Alexa Fluor 488 Annexin V/Dead Cell Apoptosis Kit (Life Technologies, Paisley, UK). Cell cycle profiles were produced with FlowJo Single Cell Analysis Software version 10, with a mean of three independent experiments used for each data point.

### Western blotting

Cell samples were washed with PBS and frozen at −80 °C. Prior to electrophoresis, samples were resuspended in 3 volumes RIPA lysis buffer, centrifuged, and protein concentration of the supernatant was determined by Bradford assay. For each sample, 50 μg protein was run on a Novex NuPAGE 4–12% Bis-Tris gel (Life Technologies, USA) according to the manufacturer’s instructions. Molecular weight was determined using Bio-Rad Precision Plus Protein Dual Color Standard (Bio-Rad, USA). Samples were transferred onto Immobilon-FL Transfer Membrane (Millipore, USA), blocked using LI-COR blocking buffer (LI-COR Biosciences, USA), diluted 1:1 with PBS. The membrane was probed with mouse monoclonal antibodies to human p53 (Abcam, ab1101, 1:1,000 dilution) and PARP (BD Biosciences, 51-6639GR, 1:1,000 dilution), and rabbit monoclonal antibodies to human p21 (Abcam, ab109520, 1:1,000 dilution) and GAPDH (Abcam, ab181602, 1:1,000 dilution). Antibodies were detected with Alexa Fluor® goat anti-rabbit or anti-mouse (Invitrogen, USA, 1:10,000 dilution) and imaged using a LI-COR Odyssey Imaging System (LI-COR Biosciences, USA).

### Statistical tests

All calculations were made using Graphpad Prism version 6. IC_50_ concentrations were calculated from the mean of three independent experiments and the significance of any differences observed for IC_50_ was calculated using Student’s T test, either unpaired test on the IC_50_ values for individual repeats, or paired test on the average data for the whole curve. The radiation enhancement ratio (RER) was calculated at 10%, 1% and 0.1% survival from non-linear regression curves fitted using the α and β values calculated using the equation SF =  = e^(α*D + β*D^2).

Isobolograms were constructed in Excel following the method of Steel and Peckham^52^; defining cumulative cytotoxicity for the two agents at SF 0.01. An ‘envelope of additivity’ was defined for the two agents between a theoretical completely independent mechanism of action and a theoretical identical mechanism of action. Experimental data points from the combined treatment clonogenic survival experiments were plotted to determine any supra-additive effects.

## Additional Information

**How to cite this article**: Carter, R. *et al.* Radiosensitisation of human colorectal cancer cells by ruthenium(II) arene anticancer complexes. *Sci. Rep.* **6**, 20596; doi: 10.1038/srep20596 (2016).

## Supplementary Material

Supplementary Information
