# Supplementary material for: Radiosensitisation of human colorectal cancer cells by ruthenium(II) arene anticancer complexes
Source: Sci Rep. 2016 Feb 12;6:20596. doi: 10.1038/srep20596 (PMC4751532; doi:10.1038/srep20596)
Supplement: Supplementary Information [file srep20596-s1.pdf]

Radiosensitisation of human colorectal cancer cells by ruthenium(II) arene anticancer complexes

Supplementary Figures S1a – S3.
Supplementary Table S1

R Carter[(1)], A Westhorpe[(1)], MJ Romero[(2)], A Habtemariam[(2)], CR Gallevo[(1)], Y Bark[(1)], N Menezes[(1)], PJ Sadler[(2)*], RA Sharma[(1)*]

[(1)] CRUK-MRC Oxford Institute for Radiation Oncology, University of Oxford
[(2)] Department of Chemistry, University of Warwick

* Correspondence to RA Sharma or PJ Sadler

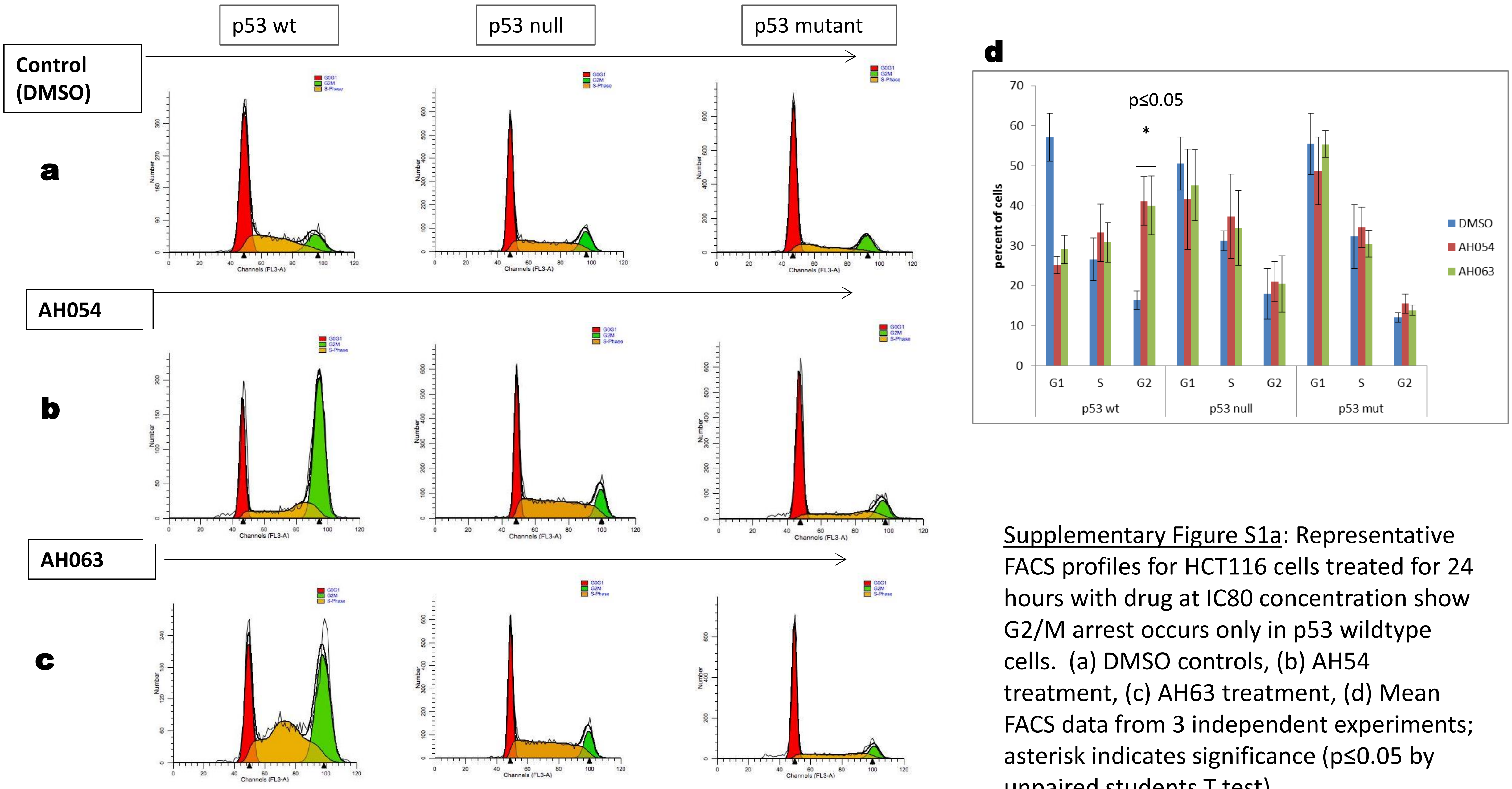

Supplementary Figure S1a: Representative FACS profiles for HCT116 cells treated for 24 hours with drug at IC80 concentration show G2/M arrest occurs only in p53 wildtype cells. (a) DMSO controls, (b) AH54 treatment, (c) AH63 treatment, (d) Mean FACS data from 3 independent experiments; asterisk indicates significance ($p \leq 0.05$ by unpaired students T test).

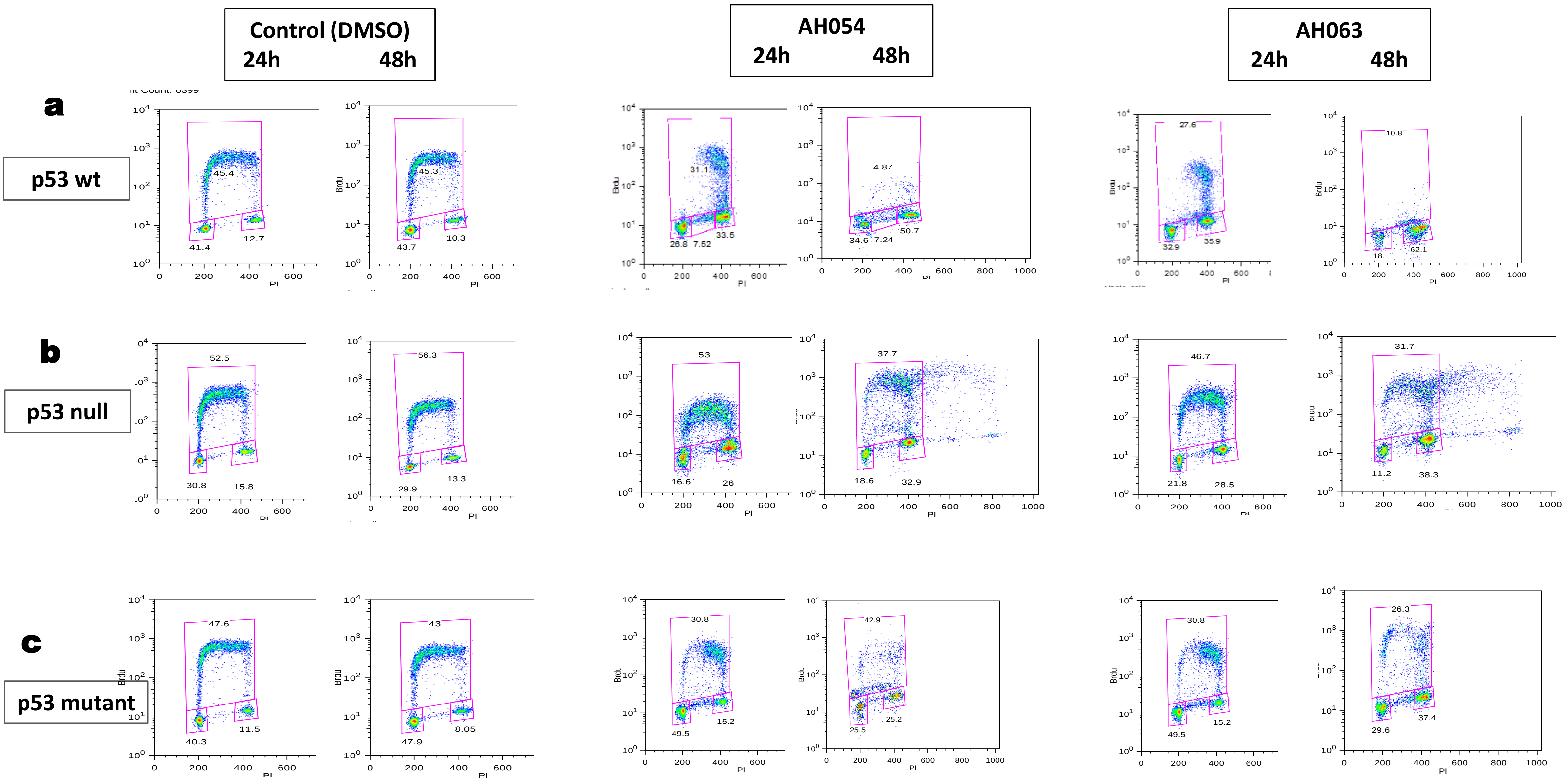

Supplementary Figure S1b: Representative FACS profiles for HCT116 cells treated with drug at IC80 concentration show G2/M arrest occurs only in p53 wildtype cells. (a) p53 wildtype, (b) p53 null, (c) p53 mutated.

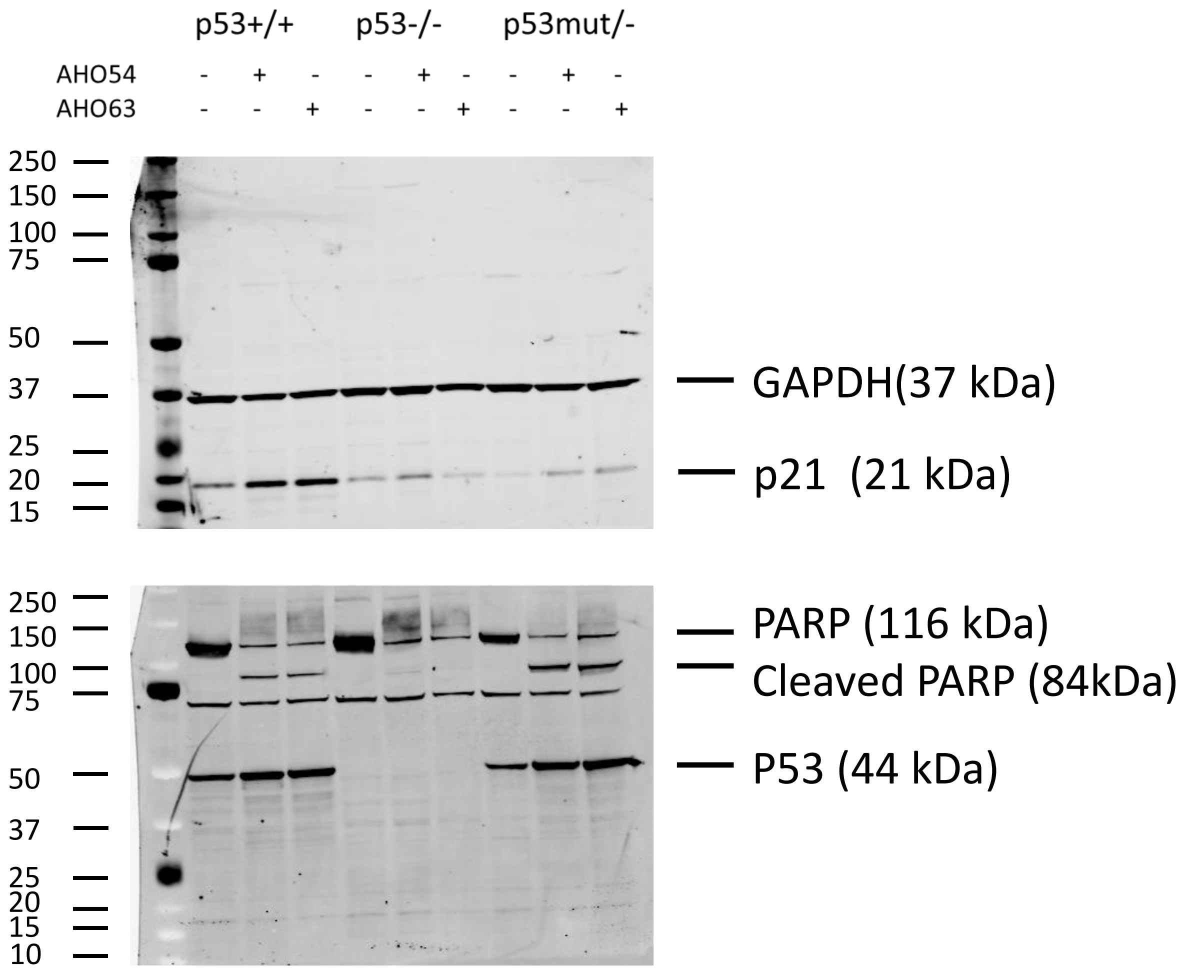

Supplementary Figure S2: Full pictures of Western blot cropped to form figure 5c. Upper panel shows rabbit primary antibodies to p21 and GAPDH, detected with Alexa Fluor® 680 conjugated goat anti-rabbit antibody. Lower panel shows the same blot with mouse primary antibodies to p53 and PARP, detected with Alexa Fluor® 790 conjugated goat anti-mouse antibody. Marker is Biorad Dual Colour prestained protein standard.

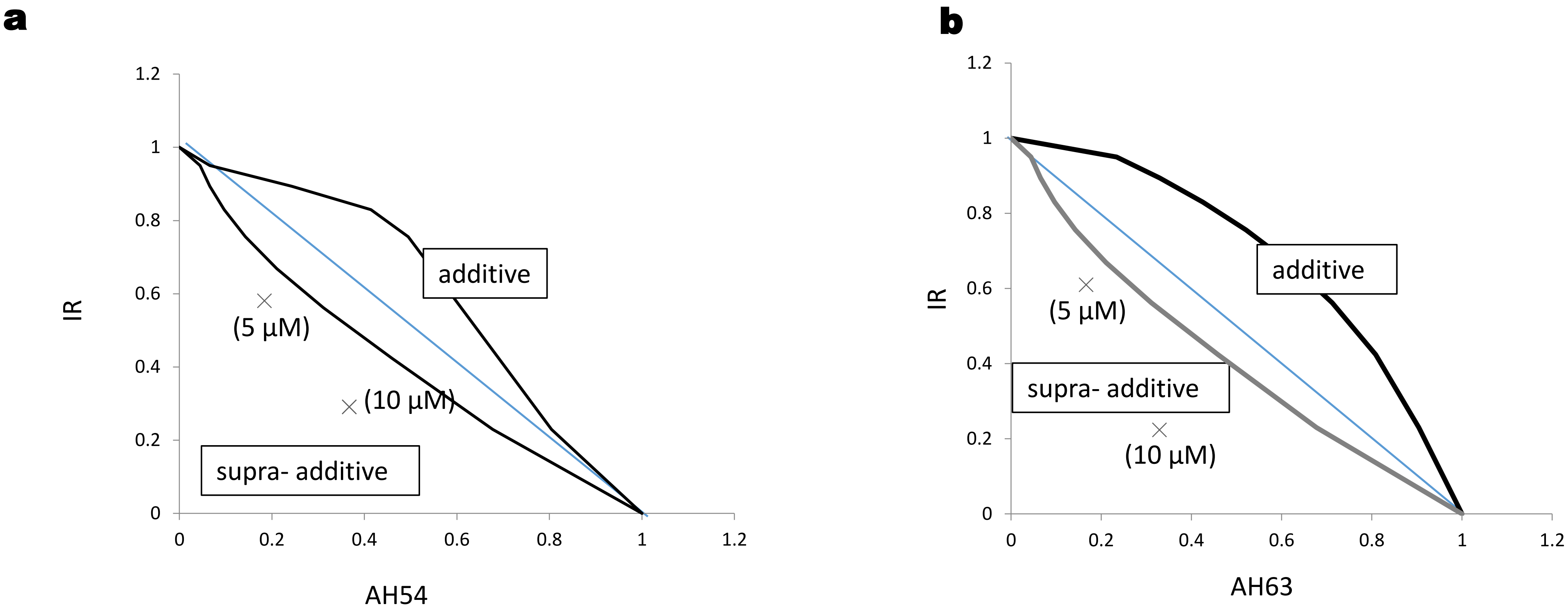

Supplementary Figure S3: Isobolograms indicating theoretical envelope of additivity (at SF 0.1) for p53 -wildtype HCT116 cells treated with combinations of ruthenium compounds and ionising radiation. Experimental data points (x) indicate clonogenic data (surviving fraction 0.1) for 5 µM or 10 µM ruthenium compound combined with radiation. The data points are all in the supra-additive region on chart (A) for AH54 and chart (B) for AH63.

Complex Code	Structure	Complex Code	Structure
AH12	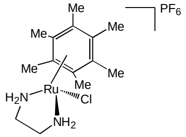	AH82	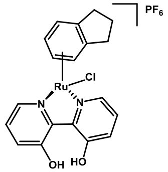
AH54	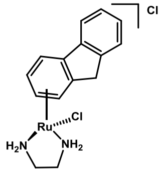	AH108	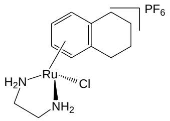
AH63	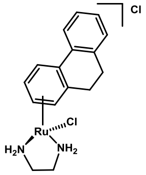	TB10	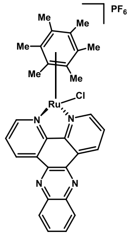
AH65	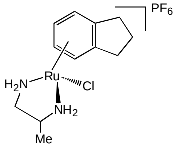	HC11	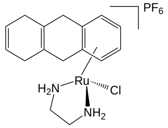
AH71	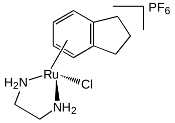	HC27	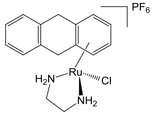
AH78	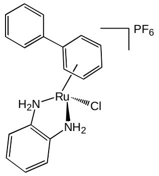	TB45	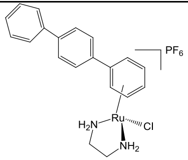
RM175	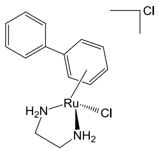	AH115	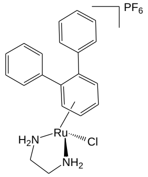

<u>Supplementary Table S1</u>: Chemical structures of the Ru(II) arene complexes tested in this project
